# Clinical characteristics, imaging phenotypes and events free survival in Takayasu arteritis patients with hypertension

**DOI:** 10.1186/s13075-021-02579-8

**Published:** 2021-07-21

**Authors:** Sun Ying, Wu Sifan, Wang Yujiao, Chen Rongyi, Huang Qingrong, Ma Lili, Chen Huiyong, Jiang Lindi

**Affiliations:** 1 Department of Rheumatology, Zhongshan Hospital, Fudan University, Shanghai, People’s Republic of China; 2 Centre of Evidence-based Medicine, Zhongshan Hospital, Fudan University, Shanghai, People’s Republic of China

**Keywords:** Takayasu arteritis, Hypertension, Hypertensive severity, Imaging phenotype, Blood pressure control status, Events free survival

## Abstract

**Background:**

Hypertension occurred in 30–80% of Takayasu arteritis (TAK) patients around the world and the occurrence of hypertension might worsen the disease prognosis. This study aimed to investigate the clinical characteristics and imaging phenotypes, as well as their associations with events free survival (EFS) in Chinese TAK patients with hypertension.

**Methods:**

This current research was based on a prospectively ongoing observational cohort-the East China Takayasu Arteritis (ECTA) cohort, centered in Zhongshan Hospital, Fudan University. Totally, 204 TAK patients with hypertension were enrolled between January 2013 and December 2019. Clinical characteristics and imaging phenotypes of each case were evaluated and their associations with the EFS by the end of August 30, 2020, were analyzed.

**Results:**

Severe hypertension accounted for 46.1% of the entire population. Three specific imaging phenotypes were identified: Cluster 1: involvement of the abdominal aorta and/or renal artery (27.5%); Cluster 2: involvement of the ascending aorta, thoracic aorta, the aortic arch, and/or its branches (18.6%); and Cluster 3: combined involvement of Cluster 1 and 2 (53.9%). Clinical characteristics, especially hypertensive severity, differed greatly among the three imaging clusters. In all, 187 patients were followed up for a median of 46 (9–102) months; 72 events were observed in 60 patients (1–3 per person). The overall blood pressure control rate was 50.8%, and the EFS was 67.9% by the end of the follow-up. Multivariate Cox regression indicated that controlled blood pressure (HR = 2.13, 95% CI 1.32–3.74), Cluster 1 (HR = 0.69, 95% CI 0.48–0.92) and Cluster 3 (HR = 0.72, 95% CI 0.43–0.94) imaging phenotype was associated with the EFS. Kaplan–Meier curves showed that patients with controlled blood pressure showed better EFS (*p* = 0.043). Furthermore, using cases with Cluster 1 imaging phenotype and controlled blood pressure as reference, better EFS was observed in patients with Cluster 2 phenotype and controlled blood pressure (HR = 2.21, 95%CI 1.47–4.32), while the case with Cluster 1 phenotype plus uncontrolled blood pressure (HR = 0.64, 95%CI 0.52–0.89) and those with Cluster 3 phenotype and uncontrolled blood pressure (HR = 0.83, 95%CI 0.76–0.92) suffered worse EFS.

**Conclusion:**

Blood pressure control status and imaging phenotypes showed significant effects on the EFS for TAK patients with hypertension.

**Supplementary Information:**

The online version contains supplementary material available at 10.1186/s13075-021-02579-8.

## Background

Takayasu arteritis (TAK) is a chronic inflammatory large-vessel vasculitis that primarily affects the aorta and its main branches [1–3]. Hypertension is a particularly important complication in patients with TAK [4–6]. According to previous reports, hypertension occurred in 33–83% of patients with TAK from different areas of the world, with younger age of disease onset [7–10]. The occurrence of hypertension could severely worsen TAK prognosis and may be a significant prognostic predictor of outcomes [11]. Furthermore, uncontrolled blood pressure was associated with a higher 5 years all-cause mortality risk than that in the healthy population, despite effective control of disease activity [12, 13]. Thus, comprehensive understanding of the disease characteristics of TAK patients with hypertension is very essential.

To our knowledge, few studies have focused on hypertension in the TAK population up to date. In a previous research, we reported that in patients with TAK-related renal artery stenosis, the prevalence of hypertension was up to 60%, with 30% refractory hypertensive cases [14]. Except renal artery stenosis, the involvement of abdominal aorta, as well as severe aortic regurgitation (AR) also could cause hypertension in TAK [15–17]. Nevertheless, data describing whether there are specific imaging features in TAK patients with hypertension as well as their associations with disease prognosis were still lacking.

Thus, this study was designed to investigate the clinical characteristics and specific imaging phenotypes of TAK patients with hypertension and to point out the associations of the clinical characteristics and imaging phenotypes with the events free survival (EFS).

## Methods

### Study design and subjects

The present study was based on a prospectively ongoing observational cohort-the East China Takayasu Arteritis (ECTA) cohort, centered in Zhongshan Hospital, Fudan University, Shanghai, China. All patients enrolled into the ECTA cohort had a confirmed diagnosis of TAK based on the 1990 American College of Rheumatology (ACR) criteria [18]. The demographic, clinical, laboratory, and treatment data were collected at baseline and each visit. The follow-up frequency was once a month in the active phase and once every 3 months in the remission phase. Disease activity was assessed using the National Institutes of Health (NIH) criteria [19]. The clinical data of all enrolled patients were recorded and stored in a unified electronic database (REDCap database system, https://redcap.zs-hospital.sh.cn).

**Fig. 1:**
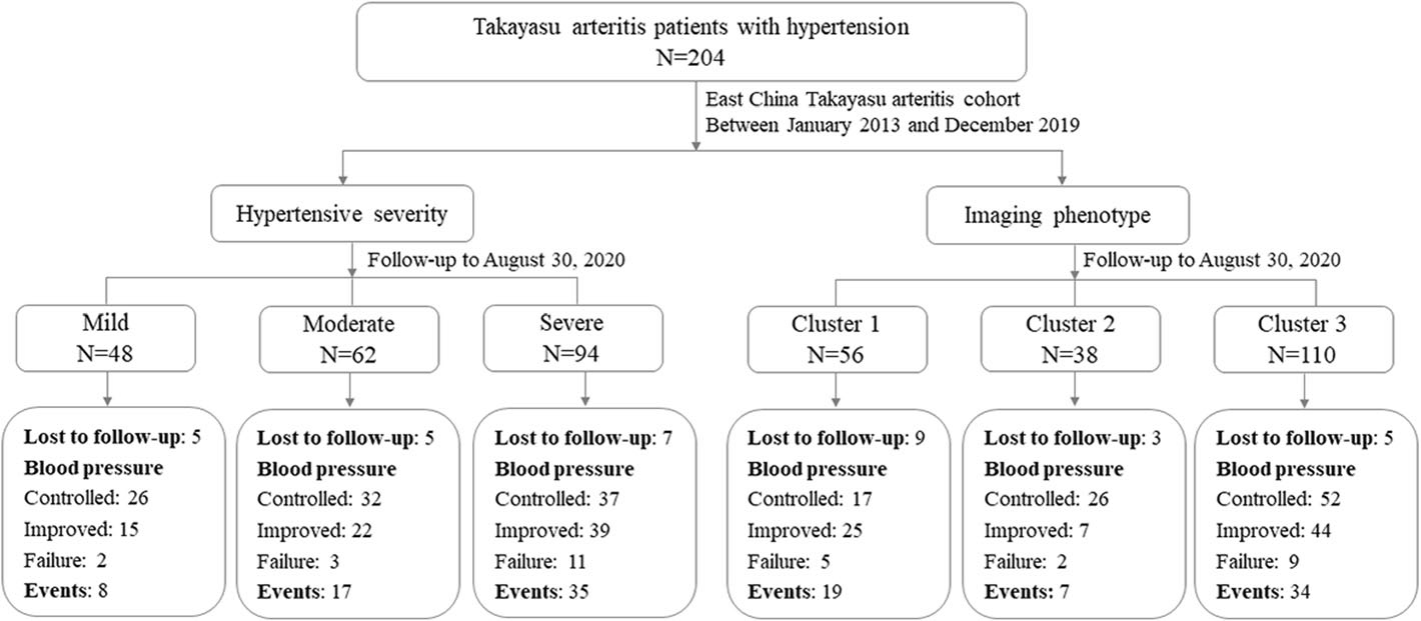
Study flow chart. In all, 204 hypertensive Takayasu arteritis patients were enrolled in to the present study from the East China Takayasu arteritis cohort between January 2013 and December 2019. Clinical characteristics and imaging features of each case were evaluated. The main outcome of the investigation was the events free survival by the end of August 30, 2020. Subgroup analysis, according to hypertensive severity and imaging phenotype, was also performed. Cluster 1: involvement of abdominal aorta and/or renal artery; Cluster 2: involvement of ascending aorta, thoracic aorta, and/or the aortic arch and its branches; and Cluster 3: combined involvement of Cluster 1 and Cluster 2.

In all, 204 TAK patients with hypertension, were enrolled in to the current research from the ECTA cohort between January 2013 and December 2019. Clinical characteristics and imaging features of each case were evaluated by professional rheumatologists. The main outcome of the investigation was the EFS by the end of August 30, 2020 (Fig. 1). Associations of the clinical and imaging features with the EFS were analyzed. The study was performed in accordance with the tenets of the Helsinki Declaration and its amendments. The study protocol was approved by the Ethics Review Board of Zhongshan Hospital (B2013-115(3)). Written informed consent was obtained from all patients.

### Blood pressure measurement

Upper-limb blood pressure was measured in all patients mainly at home. Blood pressure measurement of the four limbs and ankle brachial index (ABI) was performed in 92/204 (45.1%) patients at clinic by using a noninvasive blood pressure monitor (BP-203RPEIII, Omron Healthcare Co., Ltd., Tokyo, Japan). For those without subclavian artery involvement, the reading from the arm with the higher value was used as the reference measurement. For patients with unilateral subclavian artery involvement, the reading from the unaffected side was analyzed, while for those with bilateral subclavian artery involvement, higher values of blood pressure of the lower limbs were used for analysis.

### Classification of hypertensive severity and blood pressure control status

The severity of hypertension was classified at baseline as previously reported [20]: (i) mild: a brachial pressure of 140–159 mmHg systolic or 90–99 mmHg diastolic; (ii) moderate: a brachial pressure of 160–179 mmHg systolic or 100–109 mmHg diastolic; and (iii) severe: a brachial pressure of ≥ 180 mmHg systolic or ≥ 110 mmHg diastolic. The blood pressure should satisfy the standard in at least 20 days of a month.

Blood pressure control status was classified as: (i) controlled: systolic blood pressure (SBP) < 140 mmHg and diastolic blood pressure (DBP) < 90 mmHg; (ii) improved: SBP≥ 140 mmHg, but decreased by ≥ 20 mmHg and/or DBP ≥ 90 mmHg, but decreased by ≥ 10 mmHg; and (iii) failed: failed meeting the above-defined criteria [21]. The blood pressure should achieve the relative standard in 20 days per month for at least 3 months. Refractory hypertension was defined as a brachial pressure ≥ 160 mmHg systolic or ≥ 90 mmHg diastolic pressure despite maximal doses of three antihypertensive drugs for at least 1 month [20, 21].

### Imaging measurements

Imaging assessments, mainly the whole-body enhanced magnetic resonance angiography (MRA) or computed tomography angiography (CTA), were performed at the time of enrollment. Angiography findings were classified according to the classification by Numano et al. in 1996 [22].

Diagnosis of AR was confirmed by echocardiography according to the guideline of the American Society of Echocardiography [23]. The severity of AR was evaluated by echocardiography as previously described [24, 25].

### Outcomes

Patients, who completed at least 6 months of follow-up, were included in the outcome analysis. The occurrence of any events during the follow-up included (i) renal insufficiency including new occurrence, persistent insufficiency (≥ 6 months) or deterioration of renal function (≥ 20% increase in creatinine concentration or ≥ 20% decrease in glomerular filtration rate (GFR)); (ii) persistent refractory hypertension (≥ 6 months) or malignant hypertension; (iii) congestive heart failure including new occurrence or deterioration of heart function; (iv) new occurrence of cerebrovascular events; (v) arterial dissection or rupture of aneurysms; or (vi) TAK-related death (e.g., death caused by severe arterial stenosis or aortic dissection).

### Statistical analysis

Categorical variables were summarized as counts and percentages and were compared using chi-square or Kruskal–Wallis tests. Continuous variables are presented as means ± standard deviations (SD) or as medians with interquartile range (IQR), depending on the normality of distribution, and were compared using Student’s *t* tests, Wilcoxon tests, or one-way ANOVA. For variables with significant differences among three or more groups, pair-wise comparisons were further performed using Student’s *t* tests, Wilcoxon tests, or chi-square tests.

To identify specific imaging phenotypes for hypertensive TAK patients, 14 arteries including bilateral carotid arteries, brachiocephalic trunk, bilateral subclavian arteries, aortic arch, ascending aorta, thoracic aorta, pulmonary artery, abdominal aorta, bilateral renal artery, superior mesenteric artery, and celiac axis were included in the cluster analysis by a two-step progress as described previously [26]. Tree dendograms were created to visualize cluster patterns.

Cox proportional hazards regression model was used to evaluate associations of imaging phenotypes and clinical characteristics with EFS during the follow-up by adjusting for age, sex, disease duration, disease activity, and received treatment including medications and revascularization procedures. Hazard ratios (HR) and 95% confidence intervals (CIs) were reported. The Kaplan–Meier method was used to plot the proportion distribution of EFS in the above subgroups over time with log-rank test. Statistical analyses were performed using SPSS 22.0 (IBM Corp., Armonk, NY, USA). Two-sided *p* < 0.05 was considered to indicate statistical significance.

## Results

### Patients general characteristics

In total, 204 (33%) patients (155 [76%] female) suffered from hypertension in our cohort, with dizziness/headache (88/204, 43.1%), chest distress (57/204, 27.9%), and weakness (58/204, 28.4%) as the most common manifestations. According to the 1996 classification, type V (112/204, 54.9%) was the most common imaging type, followed by type IV (44/204, 21.6%). The demographic and clinical characteristics were presented in Supplementary Table S1.

In comparison with patients without hypertension, a higher prevalence of renal insufficiency (8.8% vs. 2.2%, *p* = 0.001) and heart failure (11.8% vs. 5.8%, *p* = 0.009) was observed in patients with hypertension. Imaging types and arterial involvement significantly differed (*p* < 0.001), showing a higher prevalence of renal artery involvement (56.9% vs. 13.3%, *p* < 0.001) and abdominal aorta involvement (51.5% vs. 22.7%, *p* < 0.001) in patients with hypertension (Supplementary Table S1).

### Characteristics of patients with different hypertensive severity

Mild, moderate, and severe hypertension was observed in 48 (23.5%), 62 (30.4%), and 94 (46.1%) cases, respectively. Clinical characteristics of the three categories were summarized in Table 1. Age, sex, and disease duration were similar among the three categories. The prevalence of renal insufficiency (*p* = 0.048), renal artery involvement (*p* = 0.043), as well as blood pressure control status (*p* < 0.001) significantly differed among the three hypertension categories. Patients with severe hypertension were more likely to experience failure control of blood pressure than those with mild (12.6% vs. 4.6%, *p* < 0.001) and moderate hypertension (12.6% vs. 5.3%, *p* = 0.008), respectively.

**Table 1:** Characteristics of patients with different hypertensive severity

	Mild	Moderate	Severe	***P-*** value
**Baseline assessment**	***N*** **= 48**	***N*** **= 62**	***N*** **= 94**	
**Demography**				
Female (*n*, %)	35 (72.9%)	47 (75.8%)	71 (75.5%)	0.752
Age (years, IQR)	40 (27–49)	35 (27–46)	37 (24–48)	0.271
Disease duration (months, IQR)	24 (2–96)	34 (6–96)	24 (3–102)	0.513
**Clinical manifestation (*n*, %)**				
Systemic symptoms (fever, weakness, etc.)	19 (39.6%)	24 (38.7%)	34 (36.2%)	0.924
Neurological symptoms (headache, amaurosis, etc.)	14 (29.2%)	26 (41.9%)	55 (58.5%)	0.544
Cardiovascular symptoms (chest distress/pain, etc.)	9 (18.8%)	16 (25.8%)	32 (34.1%)	0.273
**Complications (*n*, %)**				
Renal insufficiency	2 (4.2%) ^#,^ *	5 (8.1%)	11 (11.7%)	0.048
Heart failure	4 (8.3%)	7 (11.3%)	13 (13.8%)	0.701
Cerebral infarction	2 (4.2%)	2 (3.2%)	5 (5.3%)	0.821
**Artery involvement (*n*, %)**				
Abdominal aorta	20 (41.7%)	32 (51.6%)	53 (56.4%)	0.293
Renal artery	20 (41.7%)*	28 (45.2%)^&^	68 (72.3%)	0.043
Thoracic aorta	10 (20.8%)	22 (35.5%)	32 (34.1%)	0.293
Carotid artery	13 (27.1%)	26 (41.9%)	39 (41.5%)	0.115
Subclavian artery	27 (52.1%)	33 (53.2%)	47 (50.0%)	0.744
**Echocardiography (*n*, %)**				
Severe AR	3 (6.3%)	5 (8.1%)	11 (11.7%)	0.082
**Follow-up assessment**	***N*** **= 43**	***N*** **= 57**	***N*** **= 87**	
**Blood pressure control status (*n*, %)**				
Controlled	26 (60.5%)	32 (56.1%)	37 (42.5%)	0.082
Improved	15 (34.9%)	22 (38.6%)	39 (44.8%)	0.727
Failure	2 (4.6%)*	3 (5.3%)^&^	11 (12.6%)	< 0.001
**Events (*n*, %)**				
Persistent refractory or malignant hypertension	3 (6.9%) ^#,^ *	8 (14.0%)	16 (18.4%)	0.016
Renal insufficiency	2 (4.7%)	4 (7.0%)	6 (6.9%)	0.322
Congestive heart failure	2 (4.7%)	3 (5.3%)	2 (2.3%)	0.069
**Events free survival by the end**	81.4%*	70.2%	59.8%	0.047

*AR* aortic regurgitation; *P* value: comparison among patients with different hypertensive severity; ^#^*p* < 0.05 for comparisons between patients with mild and moderate hypertension; *p < 0.05 for comparisons between patients with mild and severe hypertension; ^&^*p* < 0.05 for comparisons between patients with between patients with moderate and severe hypertension

Immunosuppressive therapy including the oral dose of glucocorticoids and the choice of immunosuppressors did not significantly differ the three hypertensive categories, while the medium kind of antihypertensive drugs and the medication choice differed. Patients with severe hypertension had higher revascularization rates than those with moderate hypertension (45.7% vs. 24.2%, *p* = 0.008) (Supplementary Table S2).

### Characteristics of patients with different imaging phenotypes

As significant differences of artery involvement were demonstrated in patients with hypertension, cluster analysis was further performed to explore new imaging phenotypes for TAK patients with hypertension (Supplementary Fig S1): Cluster 1: involvement of abdominal aorta and/or renal artery (*n* = 56, 27.5%); Cluster 2: involvement of ascending aorta, thoracic aorta, and/or the aortic arch and its branches (*n* = 38, 18.6%); and Cluster 3: combined involvement of Cluster 1 and Cluster 2 (*n* = 110, 53.9%). The clinical characteristics of patients with different imaging phenotypes were shown in Table 2.

**Table 2:** Characteristics of patients with different imaging phenotypes

	Cluster 1	Cluster 2	Cluster 3	*P*-value
**Baseline assessment**	***N*** **= 56**	***N*** **= 38**	***N*** **= 110**	
**Demography**				
Female (*n*, %)	34 (59.6%)*	30 (78.9%)	91 (82.7%)	0.007
Age (years, IQR)	27 (20–39)^#^	42 (32–54)	39 (27–49)	< 0.001
Disease duration (months, IQR)	12 (3–56)	33 (4–72)	30 (4–120)	0.334
**Clinical manifestation (*n*, %)**				
Systemic symptoms (fever, weakness, etc.)	15 (26.8%)	17 (44.7%)	45 (40.9%)	0.320
Neurological symptoms (headache, amaurosis, etc.)	17 (30.3%)	21 (55.3%)	51 (46.4%)	0.461
Cardiovascular symptoms (chest distress/pain, etc.)	10 (17.9%)^#^	16 (42.1%)	31 (28.2%)	0.010
**Complications (*n*, %)**				
Renal insufficiency	5 (8.9%)^#^	1 (2.6%)^&^	12 (10.9%)	0.046
Heart failure	1 (1.8%)^#,^ *	5 (13.2%)	18 (16.4%)	0.013
Cerebral infarction	1 (1.8%)*	0	8 (7.3%)	0.007
**Hypertensive severity (*n*, %)**				
Moderate	18 (32.1%)	12 (31.6%)	32 (29.1%)	0.217
Severe	26 (46.4%)	10 (26.3%)^&^	58 (52.7%)	0.014
**Artery involvement (*n*, %)**				
Renal artery stenosis > 75%	29 (51.8%)*	0	40 (36.4%)	< 0.001
Abdominal aorta stenosis > 50%	19 (33.9%)	0	24 (21.8%)	< 0.001
**Echocardiography (*n*, %)**				
Severe AR	4 (7.1%)^#^	5 (11.4%)	10 (9.1%)	0.047
**Follow-up assessment**	***N*** **= 47**	***N*** **= 35**	***N*** **= 105**	
**Blood pressure control status (*n*, %)**				
Controlled	17 (36.2%)^#^	26 (74.3%)	52 (47.3%)	< 0.001
Improved	25 (53.2%)	7 (20.0%)	44 (40.0%)	0.117
Failure	5 (10.6%)^#^	2 (5.7%)	9 (8.2%)	0.032
**Events (*n*, %)**				
Persistent refractory or malignant hypertension	9 (19.1%)^#^	3 (8.6%)^&^	15 (13.6%)	0.024
Renal insufficiency	4 (8.5%)	0	8 (7.3%)	0.178
Congestive heart failure	1 (2.1%)^#^	2 (5.7%)^&^	4 (3.8%)	0.017
**Events free survival by the end**	59.6%^#^	80.0%	67.6%	0.049

Cluster 1: involvement of abdominal aorta and/or renal artery; Cluster 2: involvement of ascending aorta, thoracic aorta, aortic arch and its branches; Cluster 3: combined involvement of Cluster 1 and Cluster 2; *AR* aortic regurgitation; *P* value: comparison among patients with different imaging phenotypes; ^#^*p* < 0.05 for comparisons between patients with Cluster 1 and Cluster 2 phenotype; *p < 0.05 for comparisons between patients with Cluster 1 and Cluster 3 phenotype; and ^&^*p* < 0.05 for comparisons between patients with Cluster 2 and Cluster 3 phenotype.

**Fig. 2:**
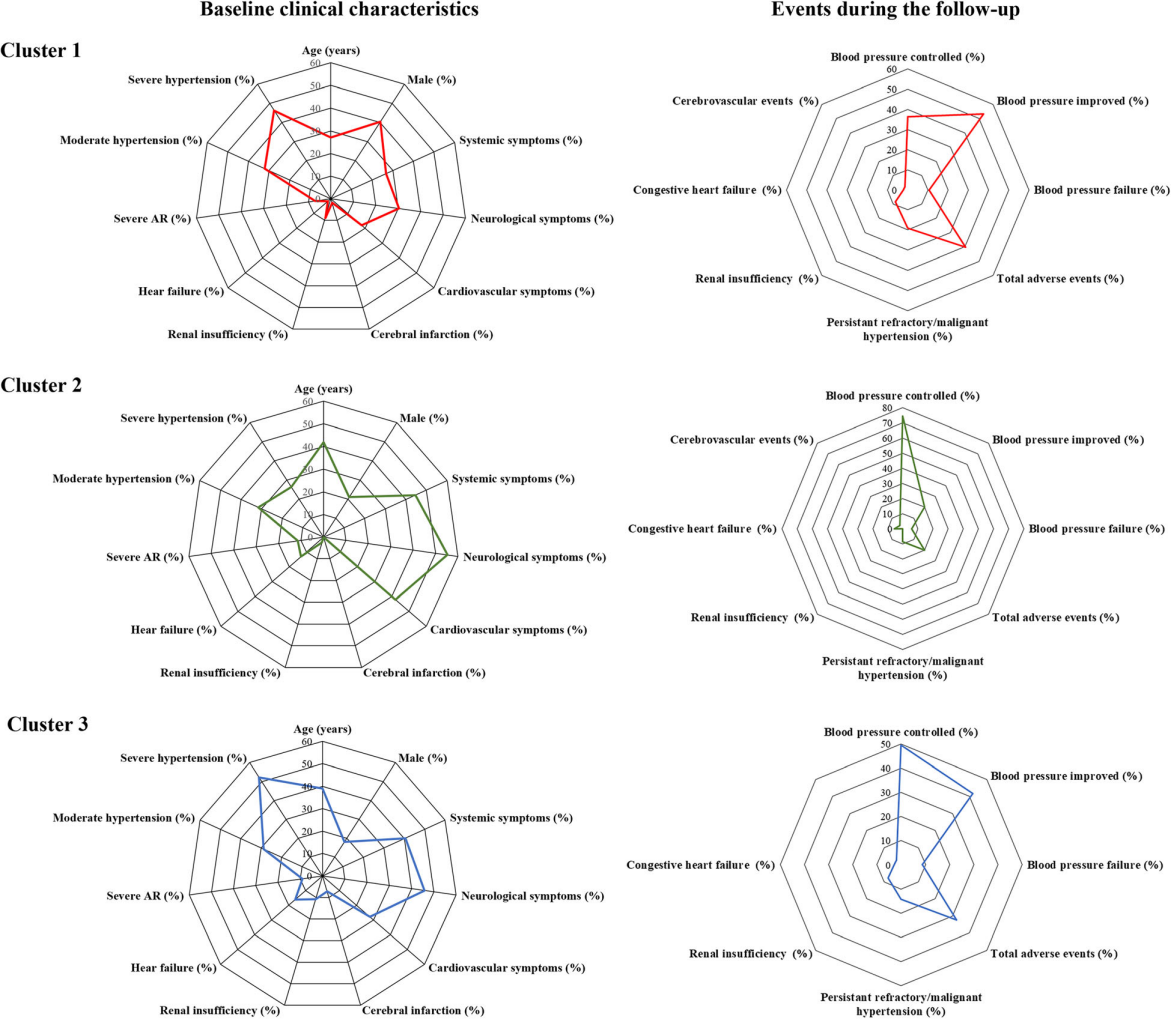
Clinical characteristics and follow-up events of patients with different imaging phenotypes. Clinical characteristics at the baseline in the radar map (left) included age, sex, clinical manifestations, and baseline complications. Blood pressure control status as well as events, including prevalence of total events, persistent refractory/malignant hypertension, renal insufficiency, congestive heart failure, and cerebrovascular events were shown in the right radar map. AR: aortic regurgitation.

Besides sex (*p* = 0.007) and age (*p* < 0.001), the prevalence of baseline features, including renal insufficiency (*p* = 0.046), heart failure (*p* = 0.013), cerebral infarction (*p* = 0.007), severe hypertension (*p* = 0.014), and severe AR (*p* = 0.047) differed significantly among the three imaging phenotype clusters. Furthermore, Cluster 1 had a higher prevalence of severe (> 75%) renal artery stenosis than Cluster 3 (51.8% vs. 36.4%, *p* = 0.036), though Cluster 3 involves some same lesions with Cluster 1. The blood pressure control status also differed among the three clusters of imaging phenotypes. Patients with Cluster 1 imaging phenotype experienced a lower prevalence of controlled hypertension (36.2% vs. 74.3%, *p* < 0.001) and higher prevalence of failure control (10.6% vs. 5.7%, *p* = 0.019) than those with Cluster 2 phenotype (Fig. 2). In addition, immunosuppressive and anti-hypertensive therapy did not significantly differ among patients with different imaging phenotypes (Supplementary Table S3).

### Characteristics of patients with different blood pressure control status

**Fig. 3:**
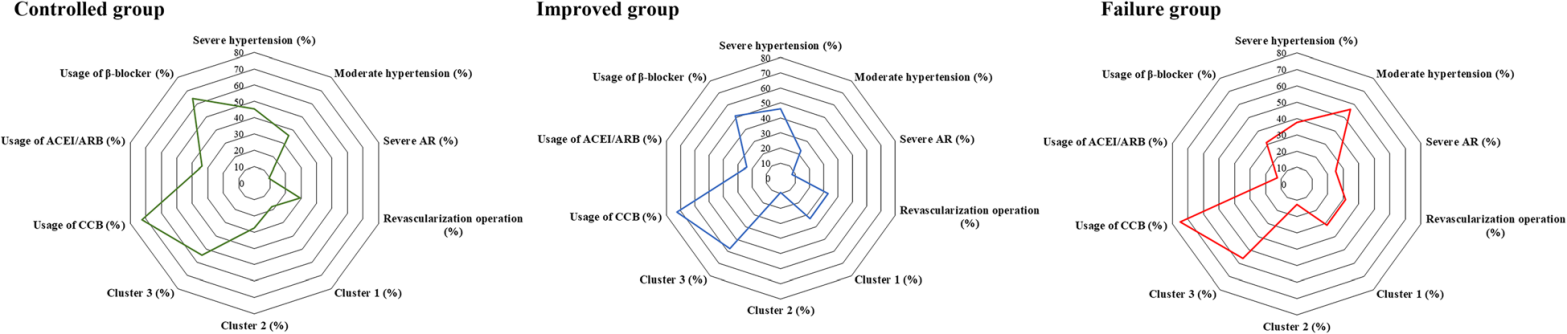
Characteristics of patients with different blood pressure control status. Characteristics showed in the radar map included imaging phenotype (cluster), hypertensive degree, severe AR, and treatment (the usage of hypertensive drugs and Revascularization operation). AR: aortic regurgitation; CCB: calcium channel blocker; ACEI/ARB: angiotensin-converting enzyme inhibitor/angiotensin receptor blocker; Cluster 1: involvement of abdominal aorta and/or renal artery; Cluster 2: involvement of ascending aorta, thoracic aorta, aortic arch, and its branches; Cluster 3: combined involvement of Cluster 1 and Cluster 2

The overall blood pressure controlled, improved, and failure rates were 50.8%, 40.6%, and 8.6%, respectively. The clinical characteristics of patients with different blood pressure control status were summarized in Table 3. The prevalence of severe AR differed among patients with different blood pressure control status (*p* < 0.001). Lower prevalence of Cluster 1 imaging phenotype was observed in the controlled group compared with improved (17.9% vs. 32.9%, *p* = 0.031) and failure group (7.9% vs. 31.3%, *p* = 0.047). Meanwhile, higher prevalence of Cluster 2 imaging phenotype was also observed in the controlled group compared with improved (27.4% vs. 9.2%, *p* = 0.001) and failure group (27.4% vs. 12.5%, *p* = 0.024) (Fig. 3).

**Table 3:** Characteristics of patients with different blood pressure control status

	Controlled ***N*** = 95	Improved ***N*** = 76	Failure ***N*** = 16	***P*** -value
**Demography**				
Female (*n*, %)	72 (75.8%)	54 (71.1%)	12 (75.0%)	0.313
Age (years, IQR)	31 (24–42)	34 (22–39)	29 (21–38)	0.411
**Hypertensive severity (*n*, %)**				
Moderate	34 (35.8%)	17 (22.4%)	5 (37.5%)	0.287
Severe	44 (45.3%)	33 (46.1%)	10 (56.3%)	0.114
**Echocardiography (*n*, %)**				
Severe AR	9 (9.5%)*	6 (7.9%)^&^	4 (25.0%)	< 0.001
**Imaging phenotype (*n*, %)**				
Cluster 1	17 (17.9%) ^#,^ *	25 (32.9%)	5 (31.3%)	0.035
Cluster 2	26 (27.4%) ^#,^*	7 (9.2%)	2 (12.5%)	< 0.001
Cluster 3	52 (54.7%)	44 (57.9%)	9 (56.3%)	0.301
**Immunosuppressive treatment**				
Glucocorticoid (prednisone, mg/day, IQR)	35 (15–40)	30 (7–40)	30 (15–40)	0.317
Cyclophosphamide (*n*, %)	20 (21.2%)	13 (17.1%)	5 (31.3%)	0.289
Leflunomide (*n*, %)	21 (22.1%)	14 (18.4%)	4 (25.0%)	0.376
Biological agents (*n*, %)	11 (11.6%)	4 (5.3%)	2 (12.5%)	0.053
**Anti-hypertensive treatment**				
Number of antihypertensive drugs (kinds, IQR)	2 (1–3)	3 (1–4)	3 (2–4)	0.074
CCB (*n*, %)	69 (72.6%)	55 (72.4%)	12 (75.0%)	0.428
ACEI/ARB (*n*, %)	32 (33.7%)*	18 (23.7%)	2 (12.5%)	0.029
β-blocker (n, %)	61 (64.2%)*	39 (51.3%)	5 (31.3%)	0.033
Diuretic (*n*, %)	23 (24.2%)	17 (22.4%)	6 (37.5%)	0.217
Clonidine (*n*, %)	4 (4.2%)*	5 (6.6%)^&^	4 (25.0%)	0.015
**Revascularization operation (*n*, %)**	28 (29.5%)	25 (32.9%)	5 (31.3%)	0.221
**Events free survival by the end**	71.8%*	65.8%	50.0%	0.041

*AR* aortic regurgitation; Cluster 1: involvement of abdominal aorta and/or renal artery; Cluster 2: involvement of ascending aorta, thoracic aorta, aortic arch, and its branches; Cluster 3: combined involvement of Cluster 1 and Cluster 2; CCB: calcium channel blocker; ACEI/ARB: angiotensin-converting enzyme inhibitor/angiotensin receptor blocker; *p* value: comparison among patients with different blood pressure control status; ^#^*p* < 0.05 for comparisons between patients with controlled and improved hypertension; *p < 0.05 for comparisons between patients with controlled and failed controlled hypertension; ^&^*p*< 0.05 for comparisons between patients with improved and failed controlled hypertension

What is more, immunosuppressive treatments did not significantly differ among patients with different blood pressure control status, while anti-hypertensive treatment differed greatly. Patients in the controlled group showed more usage of angiotensin-converting enzyme inhibitor/angiotensin receptor blocker (ACEI/ARB) (33.7% vs. 12.5%, *p* < 0.001) and β-blocker (64.2% vs. 31.3%, *p* = 0.022) compared with the failure group (Fig. 3).

### Events free survival by the end of the follow-up

Totally, 187/204 (91.7%) patients were followed up for a median of 46 (9–102) months, among whom, 127 cases did not experience any events, with a medium follow-up duration of 36 (8–100) months. Seventy-two events (1–3 per person) were observed in 60 (32.1%) patients, with a median follow-up duration of 48 (6–92) months. The events included persistent refractory or malignant hypertension (27, 14.4%), persistent or deteriorated renal insufficiency (12, 6.4%), heart dysfunction (7, 3.7%), cerebrovascular events (5, 2.7%), aortic dissection (2, 1.1%), abdominal aortic dissection (2, 1.1%), rupture of abdominal aortic aneurysm (1, 0.5%), and death (4, 2.1%: two due to heart dysfunction, one due to rupture of aortic dissection, and one due to cerebrovascular event after bypass surgery of thoracic and abdominal aorta).

The EFS by the end of the follow-up was 67.9% in the entire population, while it was 81.4%, 70.8%, and 59.8% in patients with mild, moderate, and severe hypertension, respectively; 59.6%, 80.0%, and 67.6% in patients with Cluster 1, Cluster 2, and Cluster 3 imaging phenotype, respectively; and 75.8%, 61.8%, and 50.0% in patients with controlled, improved, and control failure hypertension, respectively (Tables 1 and 2).

### Associations of the clinical characteristics and imaging phenotypes with events free survival

Multivariate Cox regression analysis, with adjustments of age, sex, disease duration, disease activity, and received medications, indicated that co-existence of severe AR (HR = 0.87, 95%CI 0.64–0.95, *p* = 0.043), controlled blood pressure (HR = 2.13, 95%CI 1.32–3.78, *p* = 0.047), Cluster 1 (HR = 0.69, 95%CI 0.48–0.92, *p* = 0.017), and Cluster 3 imaging phenotype (HR = 0.72, 95%CI 0.43–0.94, *p* = 0.048) were significantly associated with the EFS (Table 4). In further analysis, patients, who had Cluster 1 imaging phenotype with controlled blood pressure was set as reference, cases with Cluster 2 imaging phenotype and controlled blood pressure showed better EFS (HR = 2.21, 95%CI 1.47–4.32, *p* = 0.027), while those had Cluster 1 imaging phenotype with uncontrolled blood pressure (HR = 0.64, 95%CI 0.52–0.89, *p* = 0.031) and Cluster 3 imaging phenotype with uncontrolled blood pressure (HR = 0.83, 95%CI 0.76–0.92, *p* = 0.048) suffered worse EFS (Table 4).

**Table 4:** Multivariate Cox regression analysis of risk factors associated with events free survival during the follow-up

	HR	95% CI	*P* -value
**Baseline complications**			
Renal insufficiency	0.84	0.77–3.18	0.167
Heart dysfunction	0.89	0.81–4.13	0.241
Cerebrovascular events	0.96	0.79–2.14	0.383
Co-exist with severe AR	0.87	0.64–0.95	0.043
**Imaging phenotype**			
Cluster 1 imaging phenotype	0.69	0.48–0.92	0.017
Cluster 2 imaging phenotype	1.27	0.77–4.21	0.441
Cluster 3 imaging phenotype	0.72	0.43–0.94	0.048
**Baseline hypertensive severity**			
Mild	1.79	0.78–1.32	0.108
Moderate	1.03	0.82–2.11	0.094
Severe	0.87	0.63–3.18	0.069
**Blood pressure control status**			
Controlled	2.13	1.32–3.78	0.047
Improved	1.97	0.89–2.31	0.136
Failure	0.74	0.68–1.32	0.087
**Cluster 1+ controlled blood pressure**	**1 (reference)**		
Cluster 1+ uncontrolled blood pressure	0.64	0.52–0.89	0.031
Cluster 2+ controlled blood pressure	2.21	1.47–4.32	0.027
Cluster 2+ uncontrolled blood pressure	1.48	0.89–3.11	0.074
Cluster 3+ controlled blood pressure	1.13	0.91–2.33	0.069
Cluster 3+ uncontrolled blood pressure	0.83	0.76–0.92	0.048

Cluster 1: involvement of abdominal aorta and/or renal artery; Cluster 2: involvement of ascending aorta, thoracic aorta, aortic arch, and its branches; Cluster 3: involvement of Cluster 1 plus Cluster 2; AR: aortic regurgitation; adjustment: age, sex, disease duration, as well as disease activity and treatment including medication and revascularization procedure; uncontrolled blood pressure: improved and failure blood pressure control status

**Fig. 4:**
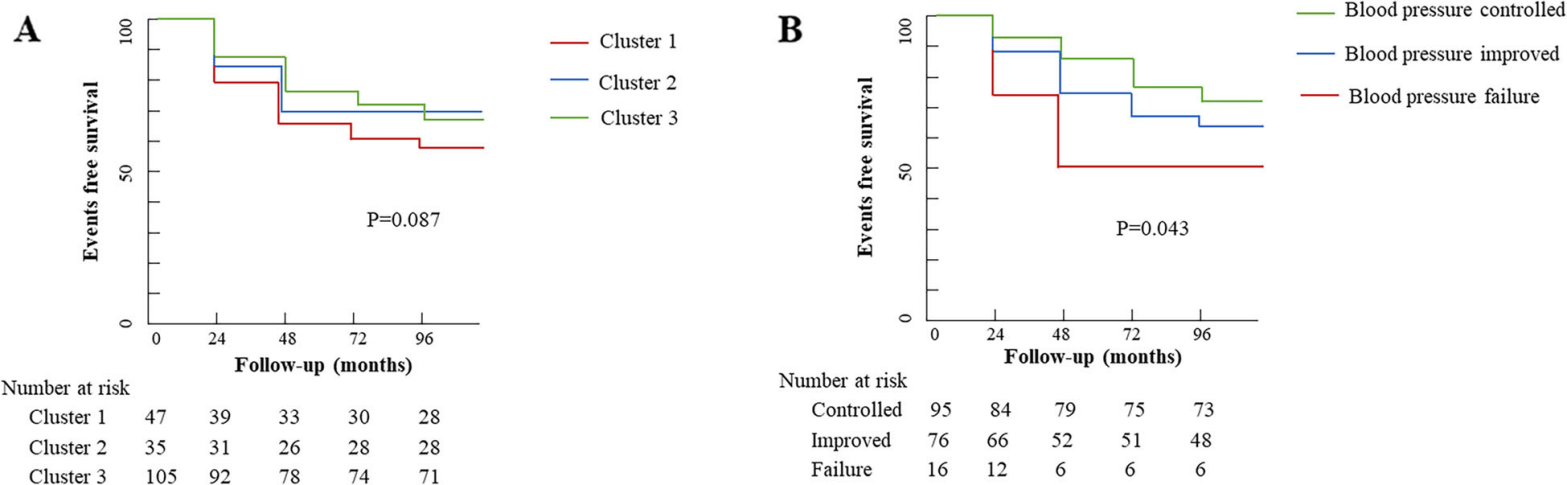
Events free survival in patients with different imaging phenotypes and with different blood control status. **A** Events free survival in patients with different imaging phenotypes. **B** Events free survival in patients with different blood pressure control status. Cluster 1: involvement of abdominal aorta and/or renal artery; Cluster 2: involvement of ascending aorta, thoracic aorta, and/or the aortic arch and its branches; and Cluster 3: combined involvement of Cluster 1 and Cluster 2.

Kaplan–Meier curves showed that patients with Cluster 1 imaging phenotype might suffer from worse EFS in comparison with Cluster 2 and Cluster 3 imaging phenotype, though this difference was not statistically significant (Fig. 4A). Patients with controlled blood pressure showed better EFS during the follow-up (Fig. 4B).

## Discussion

The present study aimed to summarize the disease characteristics of TAK patients with hypertension and highlight potential determinants to the EFS. We found that (i) about 33% of TAK patients in our cohort suffered from hypertension, among whom, almost half were severe hypertension; (ii) three specific imaging phenotypes were identified for TAK patients with hypertension, which could be distinguished from those without hypertension; (iii) only 50.8% patients got controlled blood pressure in the present study and the overall EFS was 67.9% by the end of a median 48 months follow-up; and (iv) patients with controlled hypertension showed better EFS, while imaging phenotype also showed effects on the EFS, though not statistically significant.

Previous studies have reported that hypertension occurred in 33–83% of TAK patients, with younger disease onset age (mostly < 40 years) [7–10, 21]. One former study even indicated that a combination of hypertension and elevated erythrocyte sedimentation rate (ESR) was useful for diagnosing TAK in patients < 18 years of age [27]. Our data pointed out that 33% of TAK patients suffered from hypertension, which was consistent with these previous studies. Furthermore, severe hypertension was observed in almost half of the hypertensive cases in our cohort, and severe hypertensive patients were more likely to complain of renal insufficiency and failure to control the elevated blood pressure. These findings call for physicians’ awareness of the diagnosis of TAK in young individuals presenting with hypertension, especially in those with indecipherable severe hypertension.

Previous studies have revealed that renal artery stenosis-associated hypertension was observed in about 50% of TAK cases [12, 27, 28]. In the current study, we also found that the renal artery (60%) was the most commonly involved artery in TAK patients with hypertension, and the prevalence of severe and refractory hypertension was significantly higher in patients with renal artery stenosis (data not shown), which might support the important role of renal artery stenosis in the causes of hypertension in TAK. In addition, significant differences of artery involvement were demonstrated between patients with and without hypertension, wherein it was speculated that hypertensive patients might have specific imaging phenotypes. We confirmed this by identifying three specific imaging phenotype clusters in patients with hypertension, which could be distinguished from cases without hypertension (Fig. 2). Younger age and worse disease status, especially the prevalence of severe hypertension and renal insufficiency, was observed in patients with Cluster 1 imaging phenotype. What is more, the imaging phenotypes defined in our study also showed significant effects on the EFS. The EFS was significantly lower in Cluster 1 (59.6%) than that in Cluster 2, but similar to that in Cluster 3, which may be related to the higher prevalence of renal insufficiency and persistent refractory and/or malignant hypertension, as well as the lower prevalence of blood pressure control in Cluster 1 and Cluster 3. In addition, although renal and abdominal aorta involvement was indicated both in Cluster 1 and Cluster 3, Cluster 1 had a higher prevalence of severe (> 75%) renal artery stenosis than Cluster 3. Future studies would be needed to determine whether poor prognosis is mainly attributed to this involvement.

Except for renal artery, hypertension in TAK could be caused by multifactorial conditions. In Cluster 2, hypertension might be caused by the involvement of the ascending aorta, thoracic aorta, aortic arch, and its branches instead of the renal and abdominal aorta. Hamida et al. reported that lesions of supraaortic trunks, carotid lesions, and immunosuppressive drugs might contribute to the genesis of hypertension in TAK [29]. Former studies have also found that dysfunctional baroreceptors are possible mechanisms involved in causing hypertension [30]. It is well recognized that a proatherogenic effect occurs in patients with TAK, which may increase arterial stiffness and decrease elasticity of arterial walls that may contribute to elevated blood pressure. In addition, severe AR was observed in 9.3% of hypertensive patients in our study, which was a little lower than that reported in a previous study [21]. Aortic regurgitation may be also associated with hypertension in TAK and is likely caused by directed valvular lesions, aneurysms arising from the aortic annulus, or annular dilation resulting from extensive dilatational changes of the ascending aorta. Furthermore, we also found that co-existence of severe AR was negatively related to the EFS. Thus, echocardiography monitoring is very necessary for the TAK population.

**Fig. 5:**
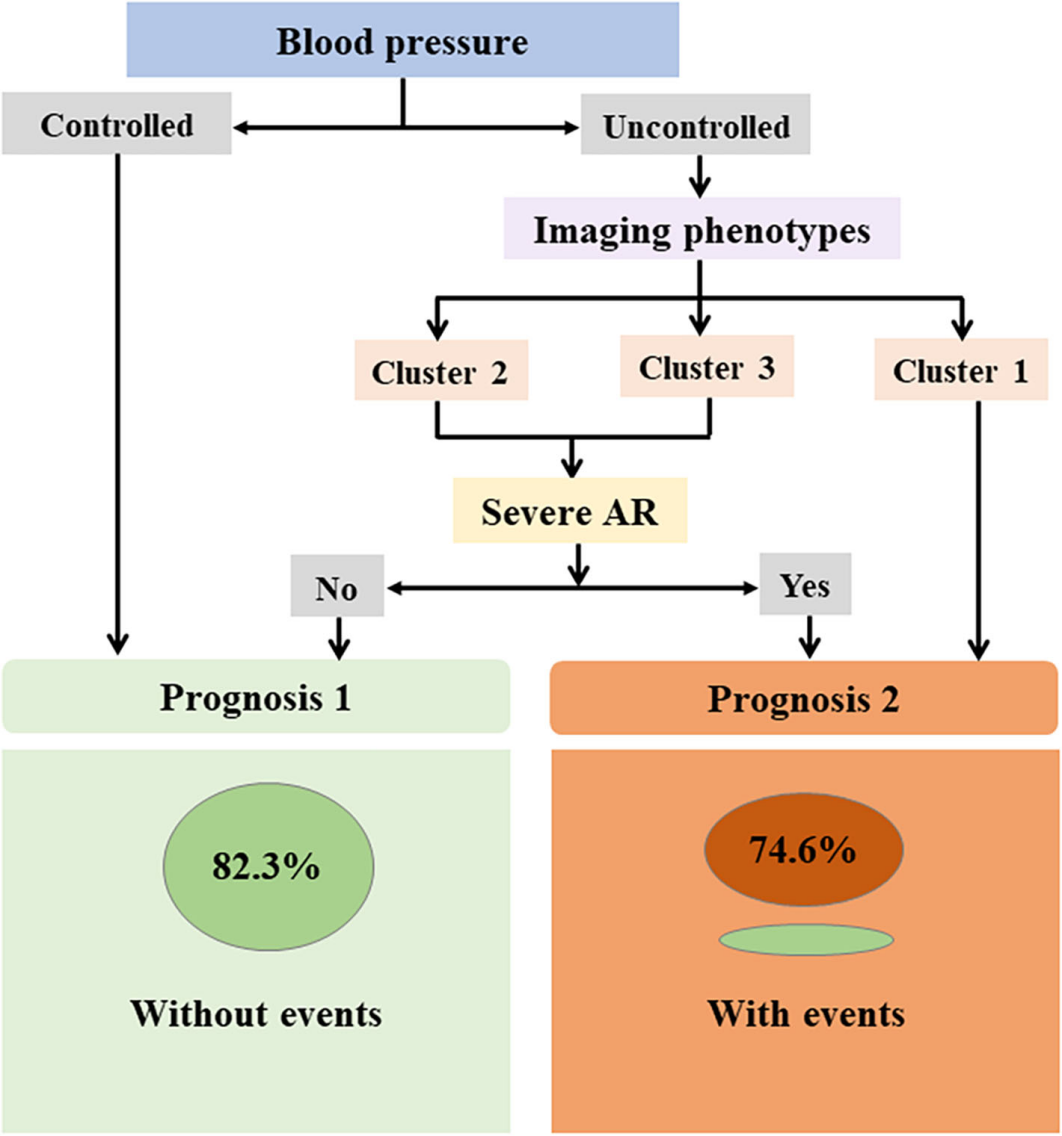
Decision tree for predicting the prognosis of Takayasu arteritis patients with hypertension. Using three variables including imaging phenotype, blood pressure control status, and co-existence of severe AR, a decision tree diagram was established to predict the disease prognosis. Through the diagram, 69.2% of patients could be classified into the right prognosis group. AR: aortic regurgitation

In the current investigation, only 50.8% of cases had blood pressure controlled during the follow-up, which was relatively low. More importantly, patients with blood pressure control showed significantly better EFS. Thus, the main treatment goal for TAK patients with hypertension should be not only to achieve and maintain disease remission, but also to achieve blood pressure control. For anti-hypertensive treatment, we found that patients with controlled blood pressure showed more usage of ACEI/ARB and β-blocker, which might indicate that ACEI/ARB and β-blocker could be a better choice for TAK patients with hypertension. However, in patients with bilateral renal artery involvement, ACEI/ARB was forbidden; in patients with unilateral renal artery involvement, close monitoring of serum creatinine and potassium should be done during the treatment with ACEI/ARB. Combined with the above data, we also made a decision tree diagram using three variables: imaging phenotype, blood pressure control status, and co-existence of severe AR (shown as Fig. 5). Through the diagram, 69.2% of patients could be classified into the right prognosis group. However, the power and accuracy of the decision tree diagram should be validated in the future, due to the small sample size of the present research.

Our study has two major limitations. First, due to the low incidence of TAK, association analyses between severity and controlled status as well as imaging phenotypic categories of hypertension with the prognosis may be underpowered, which warrants future larger studies to validate our results. Second, the follow-up duration was relatively short, and further studies with a larger sample size and longer follow-up duration are needed to validate the results.

## Conclusions

In conclusion, 33% of TAK patients suffered from hypertension in our cohort, with almost half severe cases. Three specific imaging phenotypes were identified for TAK patients with hypertension. The blood pressure control rate was 50.8%, with an overall EFS of 67.9% by the end of the follow-up. Our data support blood pressure control status and specific imaging phenotypes showed significant effects on EFS for hypertensive TAK patients.

## Supplementary Information

**Additional file 1:.** Fig S1. Tree dendogram for involved arteries of hypertensive and non-hypertensive Takayasu arteritis. Fourteen arteries including bilateral carotid arteries, brachiocephalic trunk, bilateral subclavian arteries, aortic arch, ascending aorta, thoracic aorta, pulmonary artery, abdominal aorta, bilateral renal artery, superior mesenteric artery and celiac axis were included in the cluster analysis by a two-step progress to identify imaging phenotypes for hypertensive population. Three specific imaging phenotype clusters was identified for hypertensive patients (A), which could be distinguished from non-hypertensive cases (B).

**Additional file 2:.** Supplementary Table 1. General characteristics in Takayasu arteritis patients with and without hypertension.

## Data Availability

The datasets used and/or analyzed during the current study are available from the corresponding author on reasonable request.

## Abbreviations

TAK: Takayasu arteritis
AR: Aortic regurgitation
EFS: Events free survival
ECTA: East China Takayasu arteritis
ACR: American College of Rheumatology
ABI: Ankle brachial index
SBP: Systolic blood pressure
DBP: Diastolic blood pressure
MRA: Magnetic resonance angiography
CTA: Computed tomography angiography
GFR: Glomerular filtration rate
SD: Standard deviations
IQR: Interquartile range
HR: Hazard ratio CI Confidence interval
CCB: Calcium channel blocker
ACEI/ARB: Angiotensin-converting enzyme inhibitor/angiotensin receptor blocker
ESR: Erythrocyte sedimentation rate
