# Supplementary figures and images for: Clinical characteristics, imaging phenotypes and events free survival in Takayasu arteritis patients with hypertension
Source: Arthritis Res Ther. 2021 Jul 21;23:196. doi: 10.1186/s13075-021-02579-8 (PMC8293580; doi:10.1186/s13075-021-02579-8)

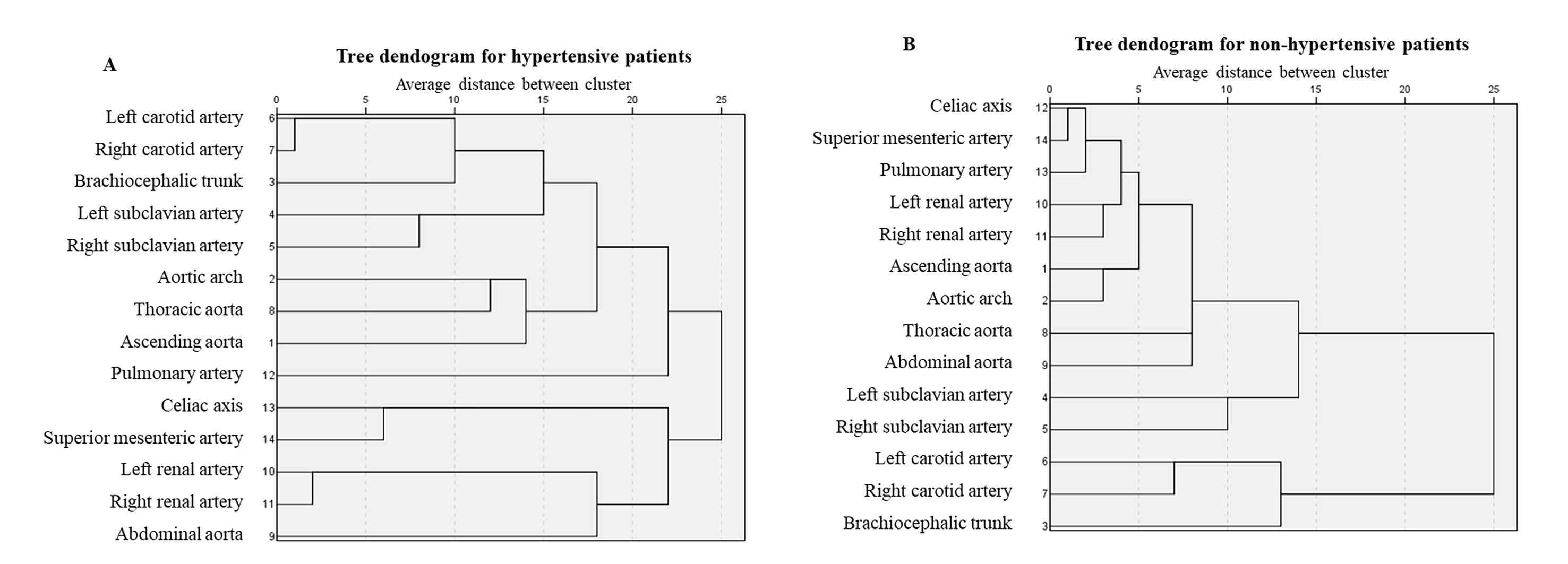

Supplement: Supplementary file 1 — Additional file 1:. Fig S1. Tree dendogram for involved arteries of hypertensive and non-hypertensive Takayasu arteritis. Fourteen arteries including bilateral carotid arteries, brachiocephalic trunk, bilateral subclavian arteries, aortic arch, ascending aorta, thoracic aorta, pulmonary artery, abdominal aorta, bilateral renal artery, superior mesenteric artery and celiac axis were included in the cluster analysis by a two-step progress to identify imaging phenotypes for hypertensive population. Three specific imaging phenotype clusters was identified for hypertensive patients (A), which could be distinguished from non-hypertensive cases (B). [file 13075_2021_2579_MOESM1_ESM.tif]
