# Supplementary material for: Clinical characteristics, imaging phenotypes and events free survival in Takayasu arteritis patients with hypertension
Source: Arthritis Res Ther. 2021 Jul 21;23:196. doi: 10.1186/s13075-021-02579-8 (PMC8293580; doi:10.1186/s13075-021-02579-8)
Supplement: Supplementary file 2 — Additional file 2:. Supplementary Table 1. General characteristics in Takayasu arteritis patients with and without hypertension. [file 13075_2021_2579_MOESM2_ESM.docx]

**Supplementary Table 1. General characteristics in Takayasu arteritis patients with and without hypertension**

|  | **Patients with hypertension**  **N=204** | **Patients without hypertension**  **N=414** | **P-value** |
| --- | --- | --- | --- |
| **Demography** |  |  |  |
| Female (n, %) | 155 (76.0%) | 354 (85.5%) | 0.001 |
| Age (years, IQR) | 37 (25-48) | 31 (23-43) | 0.002 |
| Disease duration (months, IQR) | 24 (4-96) | 12 (3-48) | 0.116 |
| **Clinical manifestation (n, %)** |  |  |  |
| Dizziness/headache | 88 (43.1%) | 156 (37.7%) | 0.192 |
| Chest distress/pain | 57 (27.9%) | 79 (19.1%)) | 0.012 |
| Amaurosis | 7 (3.4%) | 60 (14.5%) | 0.001 |
| Claudication | 13 (6.4%) | 19 (4.6%) | 0.347 |
| **Complications (n, %)** |  |  |  |
| Renal insufficiency | 18 (8.8%) | 9 (2.2%) | 0.001 |
| Heart failure | 24 (11.8%) | 24 (5.8%) | 0.009 |
| Cerebral infarction | 9 (4.4%) | 23 (5.6%) | 0.547 |
| **Physical signs (n, %)** |  |  |  |
| **Vascular murmur** |  |  |  |
| Neck area | 54 (26.5%) | 125 (30.2%) | 0.242 |
| Supra and inferior clavicular area | 26 (12.7%) | 50 (12.1%) | 0.435 |
| Renal area | 19 (9.3%) | 9 (2.2%) | <0.001 |
| Upper abdomen area | 33 (16.2%) | 22 (5.3%) | <0.001 |
| Heart valve area | 28 (13.7%) | 27 (6.5%) | 0.124 |
| **Pulseless** |  |  |  |
| Radial artery | 39 (19.1%) | 129 (31.1%) | 0.002 |
| Pedis dorsalis artery | 27 (13.3%) | 38 (9.1%) | 0.098 |
| **Lab test** |  |  |  |
| BUN (mmol/L, IQR) | 4.9 (4-6.9) | 4.4 (3.5-5.5) | 0.002 |
| SCr (µmol/L, IQR) | 64 (53-82) | 56 (48-66) | <0.001 |
| pro-BNP (pg/mL, IQR) | 212 (78-929) | 68 (33-244) | <0.001 |
| **Imaging type (n, %)** |  |  | <0.001 |
| Type I | 20 (9.8%) | 143 (34.5%) |  |
| Type II | 20 (9.8%) | 88 (21.3%) |  |
| Type III | 8 (3.9%) | 19 (4.5%) |  |
| Type IV | 44 (21.6%) | 26 (6.4%) |  |
| Type V | 112 (54.9%) | 138 (33.3%) |  |
| **Artery involvement (n, %)** |  |  |  |
| Abdominal aorta | 105 (51.5%) | 94 (22.7%) | <0.001 |
| Renal artery | 116 (56.9%) | 55 (13.3%) | <0.001 |
| Thoracic aorta | 64 (31.4%) | 104 (25.1%) | 0.213 |
| Carotid artery | 78 (38.2%) | 244 (58.9%) | <0.001 |
| Subclavian artery | 107 (52.8%) | 189 (45.7%) | 0.357 |
| **Echocardiography (n, %)** |  |  |  |
| Severe aortic AR | 19 (9.3%) | 31 (7.5%) | 0.481 |
| **Immunosuppressive treatment** |  |  |  |
| Glucocorticoid (prednisone, mg/day, IQR) | 30 (15-40) | 30 (15-40) | 0.771 |
| Cyclophosphamide (n, %) | 43 (21.1%) | 86 (20.8%) | 0.930 |
| Methotrexate (n, %) | 14 (6.9%) | 32 (7.7%) | 0.700 |
| Leflunomide (n, %) | 43 (21.1%) | 86 (20.8%) | 0.930 |
| Mycophenolate mofetil (n, %) | 10 (4.9%) | 16 (3.9%) | 0.546 |
| Azathioprine (n, %) | 12 (5.9%) | 17 (4.1%) | 0.327 |
| Biological agents (n, %) | 17 (8.3%) | 30 (7.2%) | 0.259 |

BUN: blood urea nitrogen; SCr: serum creatinine; BNP: brain natriuretic peptide; Imaging types: type I, branches of the aortic arch; IIa, ascending aorta, aortic arch, and its branches; IIb, ascending aorta, aortic arch, its branches, and thoracic descending aorta; III, thoracic descending aorta, abdominal aorta, and/or renal arteries; IV, abdominal aorta and/or renal arteries; V, combined features of IIb and IV; AR: aortic regurgitation; p-value: comparison between patients with and without hypertension, p < 0.05 indicated significance.

**Supplementary Table 2. Treatment for patients with different hypertensive severity**

|  | **Mild**  **(N=48)** | **Moderate**  **(N=62)** | **Severe**  **(N=94)** | **P-value** |
| --- | --- | --- | --- | --- |
| **Immunosuppressive treatment** |  |  |  |  |
| Glucocorticoid (prednisone, mg/day, IQR) | 20 (5-40) | 30 (7-40) | 30 (15-40) | 0.429 |
| Cyclophosphamide (n, %) | 5 (10.4%) | 13 (21.0%) | 25 (26.6%) | 0.083 |
| Methotrexate (n, %) | 2 (4.2%) | 6 (9.7%) | 6 (6.4%) | 0.511 |
| Leflunomide (n, %) | 7 (14.6%) | 15 (24.2%) | 21 (22.3%) | 0.436 |
| Mycophenolate mofetil (n, %) | 2 (4.2%) | 3 (4.8%) | 5 (5.3%) | 0.956 |
| Azathioprine (n, %) | 2 (4.2%) | 3 (4.8%) | 7 (7.4%) | 0.674 |
| Biological agents (n, %) | 2 (4.2%) | 4 (6.5%) | 11 (11.7%) | 0.047 |
| **Antihypertensive treatment** |  |  |  |  |
| Number of antihypertensive drugs (kinds, IQR) | 1 (1-2)* | 2 (2-3)^&^ | 3 (2-4) | <0.001 |
| CCB (n, %) | 29 (60.4%) | 46 (74.2%) | 79 (84.0%) | 0.088 |
| ACEI/ARB (n, %) | 12 (25.0%) | 11 (17.7%)^&^ | 39 (41.5%) | 0.005 |
| β-blocker (n, %) | 6 (12.5%)^#,^ * | 40 (64.5%) | 74 (78.7%) | <0.001 |
| Diuretic (n, %) | 0 | 11 (17.7%)^&^ | 44 (46.8%) | <0.001 |
| Clonidine (n, %) | 1 (2.1%)* | 0 | 12 (12.8%) | 0.002 |
| **Revascularization operation (n, %)** | 0 | 15 (24.2%)^&^ | 43 (45.7%) | 0.008 |

CCB: calcium channel blocker; ACEI/ARB: angiotensin converting enzyme inhibitor/ angiotensin receptor blocker; p-value: comparison among patients with different hypertensive severity; #: p<0.05 for comparisons between patients with mild and moderate hypertension; *: p<0.05 for comparisons between patients with mild and severe hypertension; &: p<0.05 for comparisons between patients with moderate and severe hypertension.

**Supplementary Table 3. Treatment for patients with different imaging phenotypes**

|  | **Cluster 1**  **N=56** | **Cluster 2**  **N=38** | **Cluster 3**  **N=110** | **P-value** |
| --- | --- | --- | --- | --- |
| **Immunosuppressive treatment** |  |  |  |  |
| Glucocorticoid (prednisone, mg/day, IQR) | 20 (10-40) | 30 (15-40) | 30 (10-40) | 0.824 |
| Cyclophosphamide (n, %) | 14 (25.0%) | 8 (21.1%) | 21 (19.1%) | 0.660 |
| Methotrexate (n, %) | 6 (10.7%) | 2 (5.3%) | 6 (5.5%) | 0.410 |
| Leflunomide (n, %) | 8 (14.0%) | 9 (23.7%) | 26 (23.6%) | 0.342 |
| Mycophenolate mofetil (n, %) | 5 (8.9%) | 0 | 5 (4.5%) | 0.144 |
| Azathioprine (n, %) | 3 (5.4%) | 3 (7.9%) | 6 (5.5%) | 0.818 |
| Biological agents (n, %) | 6 (10.7%) | 1 (2.6%) | 10 (9.1%) | 0.190 |
| **Antihypertensive treatment** |  |  |  |  |
| Number of antihypertensive drugs (kinds, IQR) | 3 (2-5) | 2 (1-2) | 2 (1-3) | 0.122 |
| CCB (n, %) | 48 (85.7%) | 22 (57.9%) | 84 (76.4%) | 0.076 |
| ACEI/ARB (n, %) | 17 (30.4%) | 15 (39.5%) | 30 (27.3%) | 0.304 |
| β-blocker (n, %) | 35 (62.5%) | 21 (55.3%) | 64 (58.2%) | 0.803 |
| Diuretic (n, %) | 17 (30.4%) | 7 (18.4%) | 31 (28.2%) | 0.452 |
| Clonidine (n, %) | 6 (10.7%) | 0 | 7 (6.4%) | 0.118 |
| **Revascularization operation (n, %)** | 18 (32.1%)^#^ | 5 (13.2%)^&^ | 35 (31.8%) | <0.001 |

Cluster 1: involvement of abdominal aorta and/or renal artery; Cluster 2: involvement of ascending aorta, thoracic aorta, aortic arch and its branches; Cluster 3: combined involvement of Cluster 1 and Cluster 2; CCB: calcium channel blocker; ACEI/ARB: angiotensin converting enzyme inhibitor/ angiotensin receptor blocker; P-value: comparison among patients with different imaging phenotypes; #: p<0.05 for comparisons between patients with Cluster 1 and Cluster 2 phenotype; *: p<0.05 for comparisons between patients with Cluster 1 and Cluster 3 phenotype; and; &: p<0.05 for comparisons between patients with Cluster 2 and Cluster 3 phenotype.
